# Functional connectivity in behavioral variant frontotemporal dementia

**DOI:** 10.1002/brb3.2790

**Published:** 2022-10-28

**Authors:** Luiz Kobuti Ferreira, Olof Lindberg, Alexander F Santillo, Lars‐Olof Wahlund

**Affiliations:** ^1^ Division of Clinical Geriatrics, Center for Alzheimer Research, Department of Neurobiology, Care Sciences and Society Karolinska Institutet Stockholm Sweden; ^2^ Centre for Psychiatry Research, Department of Clinical Neuroscience Karolinska Institutet, & Stockholm Health Care Services Stockholm Sweden; ^3^ Clinical Memory Research Unit and Psychiatry, Department of Clinical Sciences Lund University Malmö Sweden

**Keywords:** functional connectivity, fMRI, bvFTD, frontotemporal dementia, brain

## Abstract

**Introduction:**

Functional connectivity (FC)—which reflects relationships between neural activity in different brain regions—has been used to explore the functional architecture of the brain in neurodegenerative disorders. Although an increasing number of studies have explored FC changes in behavioral variant frontotemporal dementia (bvFTD), there is no focused, in‐depth review about FC in bvFTD.

**Methods:**

Comprehensive literature search and narrative review to summarize the current field of FC in bvFTD.

**Results:**

(1) Decreased FC within the salience network (SN) is the most consistent finding in bvFTD; (2) FC changes extend beyond the SN and affect the interplay between networks; (3) results within the Default Mode Network are mixed; (4) the brain as a network is less interconnected and less efficient in bvFTD; (5) symptoms, functional impairment, and cognition are associated with FC; and (6) the functional architecture resembles patterns of neuropathological spread.

**Conclusions:**

FC has potential as a biomarker, and future studies are expected to advance the field with multicentric initiatives, longitudinal designs, and methodological advances.

## Graphical abstract

Decreased FC in the Salience Network (SN) is the most consistent finding in bvFTD. The brain's functional architecture is less interconnected and efficient in bvFTD. Symptom, behavior, cognition and disease severity are associated with changes in FC.

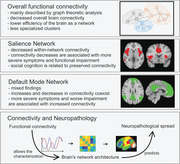

## 1 INTRODUCTION

Frontotemporal dementia (FTD) is a group of neurodegenerative diseases affecting the frontal and temporal lobes as well as subcortical structures such as the thalamus (Devenney et al., 2015; McKenna et al., 2022; WHO, 2019). FTD is the clinical syndrome, while frontotemporal lobar degeneration (FTLD) refers to neuropathological changes (Cairns et al., 2007). Behavioral variant frontotemporal dementia (bvFTD), characterized by a progressive deterioration of personality, social behavior, and cognition (Rascovsky et al., 2011), is the most common type of FTD (Lanata & Miller, 2016).

**FIGURE 1:**
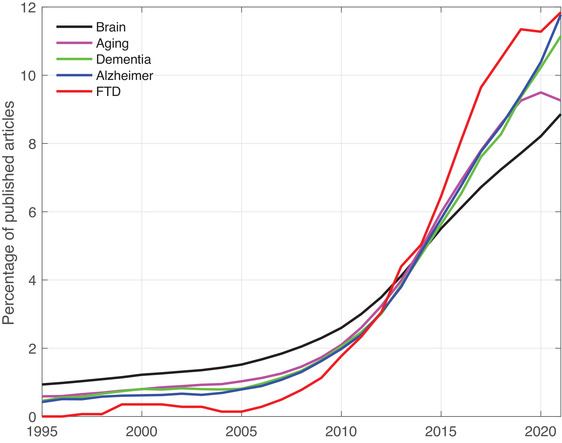
Gaining momentum: Yearly percentage of published articles about connectivity in frontotemporal dementia (FTD) and related fields. Each line represents five‐year trailing moving averages of the yearly percentage of all published articles during the period 1991–2021. Article count retrieved from PubMed (Medline) in January 2022 using the following boolean searches: for FTD, we used the boolean search (FTD OR “frontotemporal dementia”) AND brain AND connectivity; for Alzheimer's disease, Alzheimer AND brain AND connectivity; for dementia, dementia and brain and connectivity; for aging, (aging OR elderly) AND brain AND connectivity; for brain connectivity, brain AND connectivity. FTD, frontotemporal dementia

The study of brain connectivity in the field of dementia, especially in FTD is surging (Figure 1): more than half (58%) of all articles have been published in the last 5 years (Boolean search in PubMed: (FTD OR “frontotemporal dementia”) AND brain AND connectivity).

Functional connectivity (FC)—a strategy to investigate brain connectivity using neurophysiological data—is a popular tool but despite the growing literature, there is no comprehensive, in‐depth review of FC in bvFTD. Available reviews are not specifically focused (Dipasquale, 2016; Ducharme et al., 2018; Gordon et al., 2016; Irish et al., 2012; Jalilianhasanpour et al., 2019; Li Hi Shing et al., 2021; McMackin et al., 2019; Pievani, Filippini, et al., 2014; Rankin, 2020; Seeley et al., 2012; Whitwell, 2019; Zhou & Seeley, 2014) and therefore do not offer a solid summary of this field. A state‐of‐the‐art review, allowing in‐depth discussion, would consolidate current achievements and contribute to advance the research.

The focus of this review is to summarize the FC changes in bvFTD, as described in functional magnetic resonance imaging (fMRI) studies. We will briefly introduce the reader to FC, thoroughly review studies that have described FC in bvFTD, examine their findings and limitations and discuss future directions in this field.

## 2 METHODS

The main goal of this study was to perform a comprehensive narrative review of the whole literature on the field of FC in bvFTD. To this aim, we compiled relevant published articles by conducting a structured search on PubMed (https://pubmed.ncbi.nlm.nih.gov) for articles (original articles, reviews, editorials, letters) that included information about FC in bvFTD. We restricted the search for those papers involving fMRI‐derived FC. The following Boolean phrase was used: (FTD OR “frontotemporal dementia”) AND (behavioral OR behavioural) AND brain AND connectivity. Searches were performed in June 2022 (resulting in 208 published articles).

**FIGURE 2:**
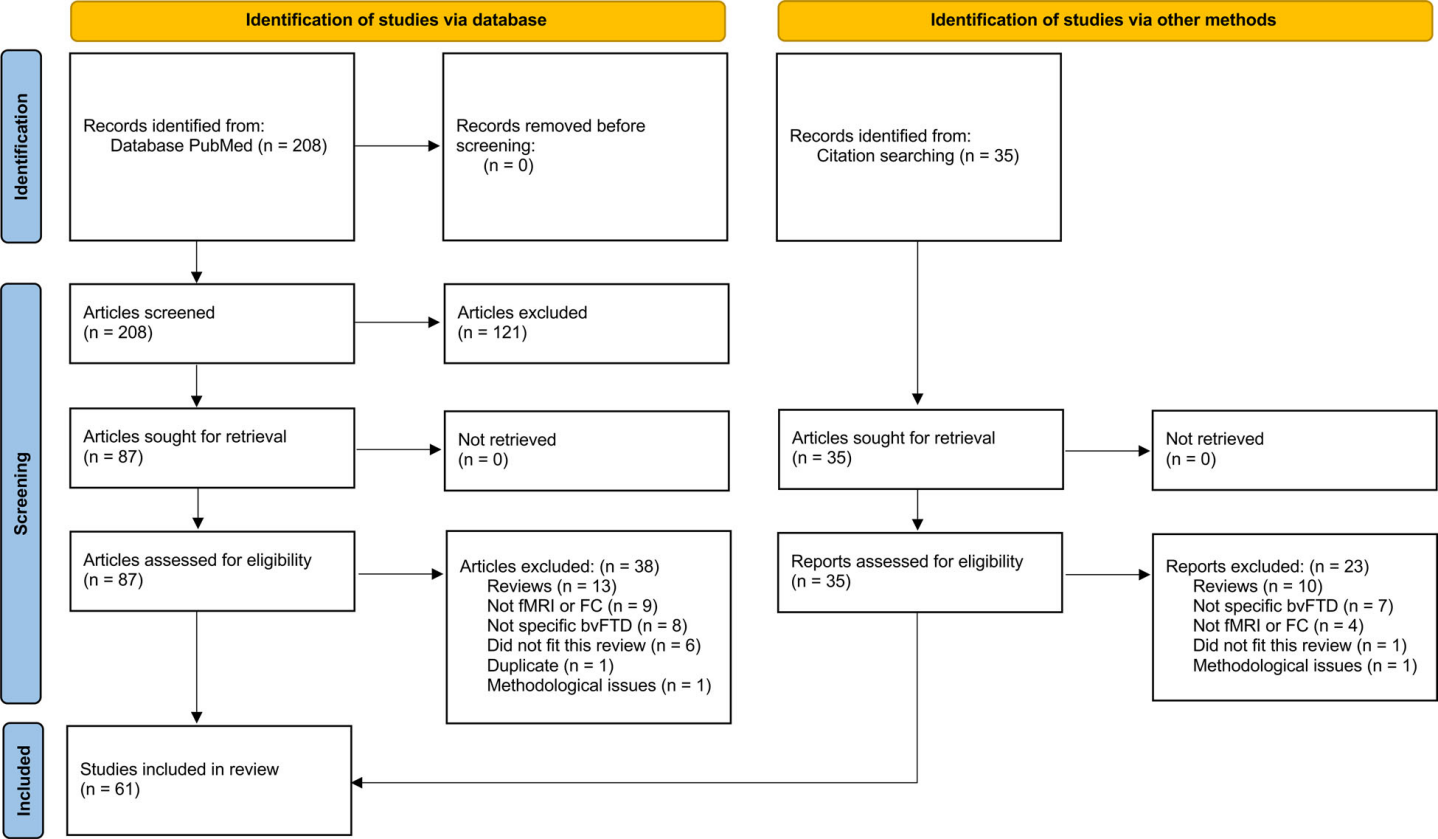
Flow diagram of the search strategy. The diagram is based on the structure of PRISMA 2020 statement (Page et al., 2021). However, the current work is a narrative review (not a systematic review). Abbreviations: bvFTD, behavioral variant frontotemporal dementia; FC, functional connectivity; fMRI, functional magnetic resonance imaging

Articles containing information about FC in bvFTD were included if the content was deemed relevant for the present review. The cited papers from these articles were also scanned, and if an article was found in this way, its references were also inspected. Figure 2 is a flow diagram describing the search, screening, and inclusion procedures.

Due to the broad nature of the present review, we chose not to perform a systematic review, which would require focused, specific research question(s) and explicit inclusion and exclusion criteria. Instead, we summarized the whole literature in this field in a narrative review. Although a meta‐analysis would be desirable, methodological heterogeneity and an intrinsic impossibility of reporting the results using standardized coordinates are obstacles. For instance, a meta‐analysis of neuroimaging in bvFTD (Kamalian et al., 2022) could only include three studies using resting‐state fMRI‐based FC (Caminiti et al., 2015; Rytty et al., 2013; Tuovinen et al., 2017).

## 3 RESULTS

### 3.1 Introducing functional connectivity

FC is the estimation of statistical relationships between neurophysiological measurements in different brain regions (Friston et al., 1993). Regions with high FC share behavioral and cognitive functions (Friston, 2011) and are thought to belong to the same network (Thomas Yeo et al., 2011).

Extracting the neural signal from two brain regions during a certain amount of time (thus recording one time series from each brain region) and then calculating the correlation coefficient between these two time series is perhaps the simplest way to understand how FC is measured. A high correlation would indicate that these two regions are highly functionally connected (Biswal et al., 1995). In practice, this “region‐to‐region approach” means (1) defining the regions to be studied and extracting the fMRI‐derived BOLD time series from each region and (2) calculating the pair‐wise correlation coefficient for each pair of regions.

The pairwise strategy based on regions‐of‐interest can be expanded to encompass the whole brain. This requires the definition of multiple regions, covering the whole brain (or the whole cortex) and then calculating the pairwise correlation between the time series from each region to each other region. As an example, this is the approach used in bvFTD by Garcia‐Cordero et al. (2021). This option is also commonly applied in studies using graph theory. In graph theory, the brain as a whole is treated as a network, while each region is called a *node* and the connections between nodes are the *edges*. In Section 3.5, we provide more details about graph theory and FC.

Alternatively, if there is a strong, focused interest in one region (or a few specific regions), it is possible to use a “seed‐based approach”: the FC between one region‐of‐interest and all the other voxels in the brain is calculated, thus building a whole‐brain “map” of FC between the region and the rest of the brain (e.g., see Sedeño et al., 2017).

The strategies above require pairwise, bivariate correlations (often, large numbers of them). A different approach is to use a multivariate technique, allowing the simultaneous analysis of the time series from all regions of the brain (commonly from all brain voxels). Multivariate approaches are data‐driven, dispensing the need for anatomical hypothesis and a priori definition of regions‐of‐interest. Independent component analysis (ICA) is a popular multivariate technique in the field of FC, with a number of available toolboxes such as FSL's MELODIC (https://fsl.fmrib.ox.ac.uk/fsl/fslwiki/MELODIC). ICA allows the delineation of groups of regions sharing similar BOLD signal by identifying a set of “components” that are maximally independent of each other (Mckeown et al., 1998). From this data‐driven approach (ICA), different networks emerge and subnetworks are often identified. For example, the DMN is frequently found decomposed into an anterior and a posterior component, and these can be further divided into smaller subnetworks (Dipasquale et al., 2015). It is possible to combine techniques, such as using ICA to identify regions that are later used as seeds for region‐based analyses, as in Whitwell et al. (2011).

There are many additional variations in how FC can be calculated and analyzed. For instance, the focus of a study may be the local connectivity throughout the brain. This would be possible by measuring “regional homogeneity”: how similar is the time series (neural activity) of a voxel compared to the signal from its neighbors (Zang et al., 2004).

There is still no consensus over the definition of the term “network” (Uddin et al., 2019). Depending on the context, the whole brain is viewed as a network, such as in graph theory (see Section 3.5). At the same time, plentiful of studies use “network” to describe a group of highly functionally connected regions (for instance when using ICA); in these cases, the brain is composed of multiple networks. This review reflects this dependence on context. In this study, the term network denotes the entire brain when referring to the functional architecture of the whole system, the “brain network” (Bullmore & Sporns, 2012). However, when a specific group of brain regions is the focus, “network” refers to a highly interconnected set (Uddin et al., 2019).

A final important methodological observation: neural activities can be positively correlated (high FC), not correlated or negatively correlated (when one region activates, the other is deactivated). This last pattern—also known as anticorrelations—is also an integral component of the brain functional architecture emerging during brain development (DeSerisy et al., 2021; Fox et al., 2005). Despite its importance, anticorrelations are often ignored or excluded from analyses (see Section 4.1.3).

FC can be calculated based on time series recorded during cognitive tasks (task‐based functional connectivity), but most FC studies rely on acquiring fMRI data without requiring the participant to perform a specific task. This is called resting state fMRI and is the next section's focus. Fact box 1 summarizes this introduction to FC.

**Table:** 

Fact box 1. Functional connectivity
‐ Functional connectivity indicates how much neural activity from different brain regions is temporally correlated.
‐ Functionally connected regions share cognitive/behavioral functions.
‐ Highly connected regions can be grouped in “networks.”
‐ A combination of high connectivity, segregation, and anticorrelations underlies the brain's functional organization.

#### 3.1.1 Resting‐state functional connectivity

**FIGURE 3:**
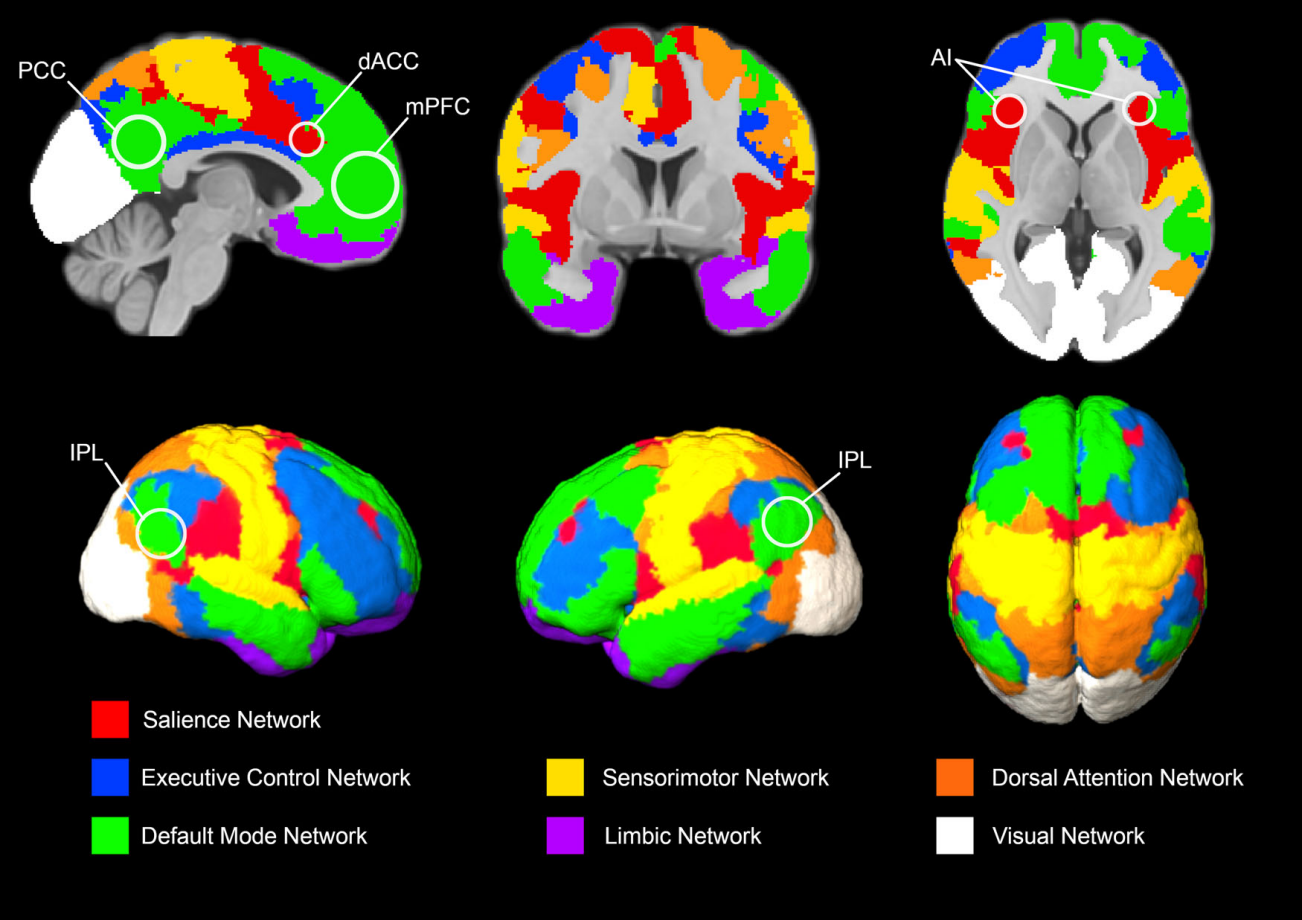
Brain networks: One atlas with seven networks. Seven brain networks derived from resting‐state fMRI data were adapted from Schaefer et al. (2018). Software used were Freeview (Freesurfer https://surfer.nmr.mgh.harvard.edu/) and MRIcroGL (https://github.com/rordenlab/MRIcroGL). Abbreviations: AI, anterior insula; dACC, dorsal anterior cingulate cortex; IPL, inferior parietal lobule; mPFC, medial prefrontal cortex; PCC, posterior cingulate cortex

In resting‐state fMRI (rs‐fMRI), data are recorded when the participant is not performing any specific task. FC can be estimated from rs‐fMRI data, resulting in resting‐state functional connectivity (RSFC). RSFC has been used to describe the functional architecture of the brain (Figure 3) (Thomas Yeo et al., 2011) and investigate brain development (Power et al., 2010), normal aging (Ferreira & Busatto, 2013), and disease‐related changes in neurological and psychiatric disorders (van den Heuvel & Hulshoff Pol, 2010). Most studies in bvFTD investigated FC using rs‐fMRI due to increased feasibility compared with task‐based fMRI.

### 3.2 The salience network and the default mode network

The two most studied networks in bvFTD are SN and the default mode network (DMN).

The DMN is the most studied network in aging (Ferreira & Busatto, 2013) and Alzheimer's disease (AD) (Badhwar et al., 2017). Its main components are the medial prefrontal cortex, the inferior parietal lobule, the hippocampus, and the posterior cingulate cortex/retrosplenial cortex/precuneus (Buckner et al., 2008; Raichle et al., 2001). The DMN is known to be active during “rest” conditions and deactivated during most attention‐demanding tasks (Raichle et al., 2001).

The SN is a group of brain regions anchored at the dorsal anterior cingulate (dACC) and the anterior insula (AI) with extensions to subcortical structures such as thalamus, hypothalamus, and amygdala (Seeley, Menon, et al., 2007). Its main role is supposed to be the detection of relevant stimuli and the coordination of neural resources, such as switching which networks are activated/deactivated in a certain moment (Uddin, 2015).

Compelling evidence implicate the SN—more specifically the insula and the anterior cingulate cortex—as harboring early neurodegenerative changes in bvFTD (Seeley, 2010). These findings in combination with the hypothesis that neurodegenerative diseases target specific large scale neural networks has led to the view that the SN is the primary target in bvFTD (Pievani et al., 2011; Seeley et al., 2009).

### 3.3 Focus on the SN and the DMN

The pioneering work by Zhou et al. (2010) demonstrated widespread decreases in RSFC within the SN in patients with bvFTD (Zhou et al., 2010). The decreased FC in the SN was to become the most common finding in the literature. On the other hand, the DMN showed a heterogeneous pattern, with both increased (the angular gyrus) and decreased RSFC (inferior and middle temporal gyrus, medial frontal orbital gyrus, and dorsolateral prefrontal cortex).

#### 3.3.1 Decreased FC in the SN is the most robust finding

A large body of work has replicated the finding of decreased FC involving the SN or its main hubs (frontoinsula and dACC) (Agosta et al., 2013; Birba et al., 2022; Borroni et al., 2012; Caminiti et al., 2015; Filippi et al., 2013; García‐Cordero et al., 2015; Lee et al., 2014; Moguilner et al., 2018; Ng et al., 2021; Rijpma et al., 2022; Sedeño et al., 2016; Sturm et al., 2018; Trojsi et al., 2015; Tuovinen et al., 2017; Whitwell et al., 2011).

The decreased FC involving the SN seems to extend beyond connections within the SN to connections between the SN and other networks and regions such as visual cortex (Agosta et al., 2013; Caminiti et al., 2015), auditory cortex (Caminiti et al., 2015), frontolimbic regions (Caminiti et al., 2015), and the left opercular part of the inferior frontal gyrus (Broca's area) (Jastorff et al., 2016).

Although decreased FC in the SN is frequently reported, there have been studies that did not find FC decreases in the SN (Canu et al., 2017; Hafkemeijer et al., 2016; Rytty et al., 2013, 2014). Moreover, studies in patients with GRN mutation and FTD found increased FC in the SN (Premi et al., 2014) and a combination of increases and decreases (Premi et al., 2016). It is possible that connectivity within SN is not homogeneously decreased (García‐Cordero et al., 2015).

#### 3.3.2 Changes in the DMN are common but results have been mixed

In the early work by Zhou et al. (2010), RSFC in the DMN is described as increased, although decreased RSFC was also found. While some subsequent work supported the view of increased RSFC in the DMN of bvFTD (Borroni et al., 2012; Farb et al., 2013; Ng et al., 2021), there is a growing body of evidence pointing toward a heterogeneous pattern in the DMN characterized by both increases and decreases (Meijboom et al., 2017; Premi et al., 2016; Trojsi et al., 2015; Whitwell et al., 2011).

Adding further weight to the ambiguous findings in the DMN, one study found only a nonsignificant trend toward decreased RSFC within the DMN (Filippi et al., 2013) and others could not find changes in the DMN (Meijboom et al., 2019; Moguilner et al., 2018).

Some evidence points toward decreased FC in the anterior part of the DMN and increased FC in the posterior DMN (Trojsi et al., 2015; Whitwell et al., 2011; Zhou et al., 2010). However, there is also a study showing an inverted pattern (increased FC anteriorly and decreased posteriorly) (Meijboom et al., 2017), others that found just increased FC in the posterior DMN (Borroni et al., 2012; Farb et al., 2013), and finally one study that found a mix of increases and decreases in both anterior and posterior DMN (Premi et al., 2016).

### 3.4 Beyond the SN and the DMN

Although the SN and, to a lesser degree, the DMN have been the major focus, a thorough review reveals that other networks and regions have also been implicated.

Decreased RSFC was found in the frontoparietal attentional network (Caminiti et al., 2015; Hafkemeijer et al., 2016; Trojsi et al., 2015), the executive network (Trojsi et al., 2015), and between the dorsal attentional network (DAN) and two other networks (DMN and executive network) (Filippi et al., 2013). The visual system presented decreases in one study (Ng et al., 2021), but another did not find significant changes in bvFTD (Moguilner et al., 2018).

Studies based on anatomical landmarks have shown that bvFTD is associated with decreased FC in the frontal, temporal and parietal lobes (Filippi et al., 2017; Reyes et al., 2018); lower regional homogeneity in the dorsolateral prefrontal cortex (DLPFC) (Farb et al., 2013); loss of negative correlation (anticorrelation) between the DLPFC and the ventromedial prefrontal cortex (Farb et al., 2013); reduced RSFC in the sensorimotor network (Lee et al., 2014; Trojsi et al., 2015); and increased RSFC in the cerebellum of GRN mutation carriers with FTD (Premi et al., 2014).

### 3.5 Graph theory in bvFTD

The brain can be understood as a network with *nodes* (discrete brain regions) and *edges* connecting the nodes. The network properties can be quantitatively described by measures from graph theory (Bullmore & Sporns, 2009). In general, studies applying graph theory in FC start by calculating pairwise correlations between time series reflecting neural activity (see Section 3.1). This results in a connectivity matrix containing the correlations between all possible pairs of network nodes (brain regions). In order to determine which nodes are “connected,” it is common to binarize this matrix by thresholding (i.e., using cutoffs so that only edges with higher values are considered “connections”). There is however no consensus on how to threshold the connectivity matrix and this is especially problematic when the goal is to describe differences between controls and a group with a disease—which is the core of most studies in bvFTD. For detailed analyses and recommendations, see van den Heuvel et al. (2017).

Graph theory studies have found that the overall connectivity (Agosta et al., 2013; Rittman et al., 2019; Sedeño et al., 2017) and network efficiency are decreased in bvFTD (Agosta et al., 2013; Saba et al., 2019; Sedeño et al., 2017). Moreover, the tendency to form specialized clusters is also decreased (Agosta et al., 2013; Sedeño et al., 2017), especially in the SN (Ng et al., 2021).

Hubs—regions presenting higher‐than‐average connection—are critical for overall network integration (Sporns, 2018). As expected, patients with bvFTD have decreased number of hubs (Rittman et al., 2019), especially in the frontal lobe (Agosta et al., 2013; Saba et al., 2019). The lack of hubs is a major contributor to the decreased efficiency of the overall network.

### 3.6 FC and neuropathology

The hypothesis that neuropathological processes spread through connected neurons was reinforced by a study that used RSFC to predict future brain atrophy (Brown et al., 2019). By combining FC and structural data, they modeled future atrophy, showing that the expansion of atrophy “bypassed some regions that were spatially adjacent but connectionally more distant from atrophied regions, such as the primary motor and sensory cortices” (Brown et al., 2019).

The view that the SN is the “target network” in bvFTD is supported by multiple lines of evidence and represents a useful and well endorsed heuristic (Seeley et al., 2009; Warren et al., 2013). However, bvFTD is more complex than just changes in the SN. As pointed by others, changes in FC occur outside the SN and the DMN (Ng et al., 2021). Additionally, findings are not restricted to within network abnormalities but comprehend a reorganization of the interplay between large‐scale networks.

### 3.7 Association with clinical variables and cognitive performance

#### 3.7.1 Clinical variables and FC

Decreased FC in the SN has been associated with more severe clinical impairment (Zhou et al., 2010) and worse neuropsychiatric symptoms (Farb et al., 2013; Lee et al., 2014). These studies also found an association between increased FC in the DMN and more severe clinical symptoms/deficits (Farb et al., 2013; Lee et al., 2014; Zhou et al., 2010). This is in line with the view that bvFTD is characterized by low connectivity in the SN and higher connectivity in the DMN.

However, it should also be noted that FC in the DMN was negatively correlated with apathy in bvFTD in one report (Trojsi et al., 2015). Moreover, the negative correlation between symptoms and FC in the SN is not found in all studies (Farb et al., 2013; Ng et al., 2021).

#### 3.7.2 Cognition and FC

Better performance on the MMSE and the Trail Making Test A has been associated with an overall increase in connectivity strength in a sample of genetic FTD (Rittman et al., 2019). An investigation of the executive domain using the Frontal Assessment Battery found that RSFC within the DMN was positively correlated to frontal lobe function (Trojsi et al., 2015).

Verbal fluency—another marker of executive functioning—has been associated with the functional organization of the brain as a network in two graph theory studies (Agosta et al., 2013; Filippi et al., 2017). These results illustrate that the overall network organization are behaviorally relevant in bvFTD.

Memory is usually not early impaired in bvFTD and therefore has been less studied. Nevertheless, one study reported a positive correlation between memory performance and RSFC between the right anterior insula and the right precuneus in symptomatic C9orf72 mutation carriers (a mix of bvFTD and ALS) (Shoukry et al., 2020).

#### 3.7.3 Social cognition and FC

RSFC in the SN is positively correlated with measures of social cognition such as interpersonal warmth (Toller et al., 2019), socioemotional sensitivity (Toller et al., 2018), and facial emotion recognition (Salamone et al., 2021).

Both the SN and the DMN play important roles in social interactions. While the SN is associated with affection and motivation in social contexts, the DMN supports social cognitive processes, including mentalizing and emotion attribution/communication (Feng et al., 2021; Mars et al., 2012). Interestingly, higher RSFC between the medial part of the anterior DMN and the dACC (a part of the SN) has been associated with better performance in social cognition (Caminiti et al., 2015).

#### 3.7.4 Other associations with FC in bvFTD

In addition to the above‐mentioned studies, there are investigations of other variables in bvFTD. For instance, reports of association between FC and mind‐wandering (O'Callaghan et al., 2019), autonomic nervous system activity (Sturm et al., 2018), heartbeat evoked potential (Birba et al., 2022), comprehension of text containing social information (Birba et al., 2021), and cognitive reserve (Premi et al., 2013). The main findings are listed in Fact box 2.

**Table:** 

Fact box 2. Main findings: Functional connectivity in behavioral variant frontotemporal dementia
‐ Decreased connectivity in the salience network (SN) is the most consistent finding.
‐ The default mode network (DMN) is also affected but there are conflicting results.
‐ Symptoms, cognition, and clinical severity have been correlated to connectivity, specially involving the SN and, to a lesser degree, the DMN.
‐ Understanding bvFTD as a disease of the SN is a well‐endorsed and useful heuristic. However, caution is advised since not all findings can be explained by this model.

### 3.8 Individual diagnosis of bvFTD using FC

Distinguishing between AD and bvFTD can be sometimes challenging. The combination of decreased FC in SN with increased FC in DMN was used in one pioneering study to correctly distinguish patients with AD from patients with bvFTD with 100% accuracy (Zhou et al., 2010).

More recently, one multicentric study managed to reach classification accuracy higher than 80% when distinguishing bvFTD against controls or from AD using only FC‐derived data (Moguilner et al., 2021). Another investigation applied graph theory to correctly classify individual participants as belonging to bvFTD or control groups (Sedeño et al., 2016).

FC can be combined with other types of data to improve classification accuracy. For instance, two studies demonstrated the superiority of combining RSFC with structural data to distinguish patients with bvFTD from controls and patients with AD (Bouts et al., 2018; Donnelly‐Kehoe et al., 2019).

Finally, one study compared FC to other measurements (gray matter, cardiac interoceptive performance, metacognition about interoception, and EEG‐derived heart‐evoked potential) (Abrevaya et al., 2020). When distinguishing bvFTD from a group of other neurological disorders (stroke, AD, and multiple sclerosis), RSFC data lead to better classification than all the other markers.

### 3.9 Genetics, FC, and bvFTD

Mutations are known to account for a significant proportion of FTD (Olszewska et al., 2016). Studies have shown that even asymptomatic mutation carriers already present changes in FC during the preclinical phase (Lee et al., 2019; Rittman et al., 2019), emerging even before brain atrophy (Lee et al., 2019).

A number of studies have found associations between FC and specific FTD‐related mutations (Lee et al., 2017; Shoukry et al., 2020; Tsvetanov et al., 2021; Whitwell et al., 2011), but there are also studies that failed to detect gene‐related changes in FC (Bouts et al., 2018; Pievani, Paternicò, et al., 2014). The influence of specific FTD‐related genes in FC is complex enough to warrant a separate review.

## 4 DISCUSSION

### 4.1 Methodological considerations

#### 4.1.1 The impact of brain parcellation

When analyzing FC with fMRI, voxels—the 3D‐analogue of a pixel—are often bundled together in regions such as the automated anatomical labeling atlas (Tzourio‐Mazoyer et al., 2002). Another alternative is to use fMRI data and analyses based on FC to identify maximally homogenous regions (parcels) (Schaefer et al., 2018). There are a number of other approaches to parcellate the brain (Eickhoff et al., 2018) but no consensus (Wig et al., 2011). Different parcellation schemes can lead to divergent results and challenges when comparing studies (Wig et al., 2011). This is not exclusive to bvFTD, and advances in the broad field of neuroimaging will hopefully be incorporated and lead to more optimal parcellation schemes in bvFTD.

#### 4.1.2 Linear and nonlinear relationships

Variables can present linear and nonlinear relationships. Linear relationships reflect a relatively preserved (constant) relationship. Nonlinear associations encompass a wider range of situations with variable rates of change. An incorrect assumption of linearity may lead to inadequate statistical methods and wrong interpretation of findings. Brain‐behavior relationships are often nonlinear. In AD, there is evidence that in some regions FC increases during the early preclinical phase and decreases sharply when amyloid deposition crosses the pathological threshold (Palmqvist et al., 2017).

**FIGURE 4:**
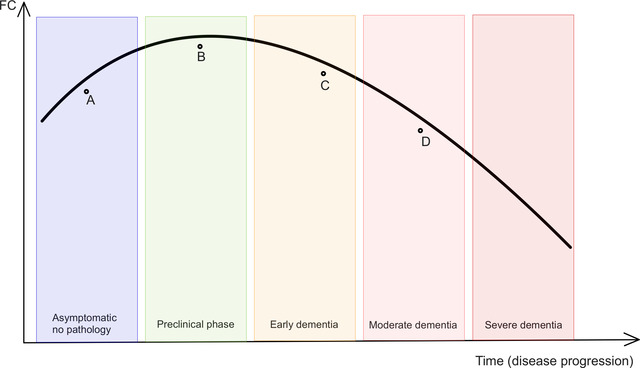
Hypothetical consequences of nonlinear relationship between functional connectivity and disease stage. Vertical axis: functional connectivity (FC). Horizontal axis: time. Cross‐sectional differences between a control group (point A) and a group in the preclinical phase—point B (mutation carrier, approaching symptom onset) will find increased FC in the preclinical group. If the same control group (point A) is compared to mild dementia (point C), there will be no significant differences. However, when the control group (point A) is compared to a more advanced dementia (point D), the decreased FC emerges

Back to FTD, two studies of GRN‐mutation carriers have provided evidence for nonlinear relationships between disease stage and RSFC (Borroni et al., 2012; Lee et al., 2019). RSFC in some brain regions increased in the stage preceding the onset of symptoms while decreasing after the development of clinical dementia, thus presenting an inverted U‐shaped curve when observed longitudinally (Figure 4).

When calculating FC, researchers measure the relationship between variables. For instance, many studies calculate FC using Pearson's correlation, which is suited to quantify linear associations. However, linear and nonlinear interactions between brain regions coexist and nonlinear methods for calculating FC have yielded higher accuracy in distinguishing bvFTD from controls (Moguilner et al., 2018, 2021). As these authors put it “the coexistence of linear and nonlinear interactions in brain connectivity is exceedingly simplified by the linearity assumption of most FC studies on neuropsychiatric diseases, which may be better characterized via nonlinear approaches” (Moguilner et al., 2018).

#### 4.1.3 Anticorrelations and positive correlations

Both positive correlations and anticorrelations are an integral part of the human brain organization (Fox et al., 2005). Anticorrelations are especially relevant in the interactions between the SN and the DMN (Seeley, Allman, et al., 2007; Uddin et al., 2009), two networks of utmost importance in the field of cognitive aging and cognitive disorders.

One challenge imposed by the coexistence of positive and negative correlations is the interpretation of “increases” and “decreases” in FC. For instance, “increased” FC is ambiguous: it may indicate that (1) a connection with positive FC has become even more positive (increased connectivity) or (2) a connection that was characterized by anticorrelation has lost its negatively correlated pattern (a loss of anticorrelations).

Many studies in the field of bvFTD focus only on positive correlations, discarding negative values (Filippi et al., 2017; Melloni et al., 2016; Moguilner et al., 2018; Ng et al., 2021; Sedeño et al., 2017; Smallwood Shoukry et al., 2020). Since the interactions between the SN and DMN are characterized by anticorrelations and because the SN and its interplay with the DMN are central in bvFTD (Seeley, Menon, et al., 2007; Zhou et al., 2010; Zhou & Seeley, 2014), excluding anticorrelations means ignoring relevant data. For instance, enhanced anticorrelation between the thalamus (a part of the SN) and the DMN was found in patients with C9orf72‐related bvFTD (Rytty et al., 2014).

#### 4.1.4 Static versus dynamic FC

Most research on FC focuses on static FC (an average measure of connectivity based on the whole time series). However, FC is dynamic: connectivity between different brain regions waxes and wanes from moment to moment (Breakspear, 2017; Hutchison et al., 2013), leading to an ever‐changing functional organization of the brain (“dynamic states”) (Calhoun et al., 2014).

The relevance of such understanding was shown by a multicentric study that found that extracting information about FC using a dynamic approach yielded higher accuracy in distinguishing individuals with bvFTD from healthy controls and AD (Moguilner et al., 2021). Dynamic FC has also been used in FTD to investigate cognitive reserve (Premi et al., 2020) and to explore GABA‐ and glutamatergic neurotransmission using transcranial magnetic stimulation (Benussi et al., 2020).

There are multiple approaches to analyze dynamic FC. Graph analysis is a popular choice. Multivariate approaches are also often employed. The field is developing rapidly and being adopted in different branches of neuroscience. On the one hand, methodological developments are rapidly evolving. On the other hand, it is very difficult to compare and synthesize results from different papers. For a methodological review, see Preti et al. (2017).

### 4.2 Limitations

#### 4.2.1 Small sample sizes

Recruiting participants with bvFTD is challenging. Sample sizes tend to be small, often varying from seven to 20 participants (Abrevaya et al., 2020; Agosta et al., 2013; Garcia‐Cordero et al., 2021; Rytty et al., 2014; Salamone et al., 2021; Sedeño et al., 2016; Smallwood Shoukry et al., 2020; Zhou et al., 2010). This leads to statistical limitations and risks (Button et al., 2013) such as not having enough statistical power. For instance, some articles had to mix patients with bvFTD and healthy controls in one group when performing correlation analyses even though it would be interesting to test for associations only in the group with bvFTD (Garcia‐Cordero et al., 2021; Salamone et al., 2021; Toller et al., 2018).

Although most studies had small samples, there are also many exceptions that succeeded in enrolling 40 or more participants (Benussi et al., 2020; Brown et al., 2019; Premi et al., 2020; Reyes et al., 2018; Saba et al., 2019). It is strongly recommended that future initiatives invest in enrolling bigger samples, which will probably require multicentric studies and data repositories (see Section 4.3.2).

#### 4.2.2 Diagnostic uncertainties and heterogeneity in bvFTD

The clinical bvFTD syndrome is diverse (Ranasinghe et al., 2016), and differential diagnosis between AD and bvFTD can be challenging (Rascovsky et al., 2011) and clinico‐pathological mismatch occurs (Whitwell, 2019). Most FC studies in bvFTD relied on clinical judgement to establish a diagnosis. Therefore, some bvFTD samples may have included patients harboring non‐FTLD pathologies such as amyloid deposition (Zhou et al., 2010).

Even if diagnosis is correct, there is still room for heterogeneity (Whitwell, 2019). Mutations in different genes seem to confer divergent patterns of brain atrophy (Whitwell et al., 2012) and connectivity (Lee et al., 2019). Different proteinopathies also have distinct patterns of gray and white matter disruption (Giannini et al., 2021) and may differentially affect the brain's networks (Warren et al., 2013). Since most studies could not verify the underlying proteinopathy, it is expected that the samples of bvFTD are mixed.

Although the prevailing view has been that bvFTD is a disease targeting the frontoinsula and the anterior cingulate cortex (Seeley, Allman, et al., 2007), different atrophy patterns have been described (Ranasinghe et al., 2016; Whitwell et al., 2009). Therefore, the view of bvFTD as a disease of the frontoinsula should be complemented by the substantial number of patients who do not fall in this category.

The results of FC studies are in accordance with these two complementary views: while there is strong support for the “frontoinsula‐anterior cingulate model,” there is also considerable within‐bvFTD heterogeneity pointing toward the need to further explore the role of genetic and neuropathological diversity in bvFTD. Therefore, it is desirable to further explore how bvFTD can be subclassified *antemortem*. This would facilitate newer analyses that—instead of being confounded by heterogeneity—take advantage of neuropathological diversity to generate new insights and progress.

#### 4.2.3 Terminology: Network taxonomy

The lack of a naming convention (Uddin et al., 2019) makes synthesizing the literature challenging. This is especially problematic to the anterior insula because it has been reported to participate in different brain networks (Uddin et al., 2019). The SN is not consistently described (Pievani et al., 2017) and can be bundled together with other regions into a group of “attentional networks” (Caminiti et al., 2015). Additionally, researchers may study bvFTD using sets of networks that do not include an explicit SN (Hafkemeijer et al., 2015) or the SN itself can be subdivided (Canu et al., 2022; Filippi et al., 2013). Finally, “considerable difficulty in detecting a robustly identifiable salience and other high cognitive networks from individual” data has been reported (Rytty et al., 2013). Even atlases of brain networks derived from big samples result in borders that share low inter‐atlas similarity, especially the DMN and SN (Doucet et al., 2019). The field of bvFTD is particularly affected by this limitation.

### 4.3 Future directions

#### 4.3.1 Diagnosis and staging

To be clinically useful, FC should be valuable for diagnosis and/or staging. Although there are studies demonstrating the potential of individual classification based on FC (see Section 3.8), FC is not close to be applied in clinical practice.

In contrast, the inclusion of FC in a framework of dynamic changes of biomarkers in bvFTD seems more attainable. The development of such framework, analogously as in AD (Jack et al., 2010), would facilitate research advancements, allowing further hypothesis testing and perhaps open the doors to biomarker‐based staging of individual patients.

Once disease‐modifying drugs are developed, it will be crucial to identify individuals at the preclinical phase who are at high risk of developing dementia. Since changes in FC seem to precede brain atrophy (Whitwell et al., 2011), it holds promise as an early biomarker. Such development will require larger samples and longitudinal studies.

#### 4.3.2 Multicentric studies and data repositories

Multicentric studies and data repositories are important strategies to address the issue of small samples and allow for greater generalization of results.

Bi‐ and multicentric studies already exist (Donnelly‐Kehoe et al., 2019; Ibañez et al., 2021; Moguilner et al., 2018; Sedeño et al., 2017), and a successful example of a multicentric initiative is the Genetic Frontotemporal Initiative (genfi.org) (Premi et al., 2019; Rittman et al., 2019; Tsvetanov et al., 2021). Another source of data is The Frontotemporal Lobar Degeneration Neuroimaging Initiative (memory.ucsf.edu/research‐trials/research/allftd).

In combination with the growing number and size of neuroimaging data repositories, a larger amount of data will allow a better understanding of FC in bvFTD, throughout its disease stages and across its genetic and neuropathological underpinnings.

## 5 CONCLUSIONS

Small samples, diagnostic uncertainties, and clinical, genetic, and neuropathological heterogeneity are the biggest challenges in the field of FC in bvFTD. Notwithstanding, the growing body of knowledge has laid the ground for the next generation of studies.

**FIGURE 5:**
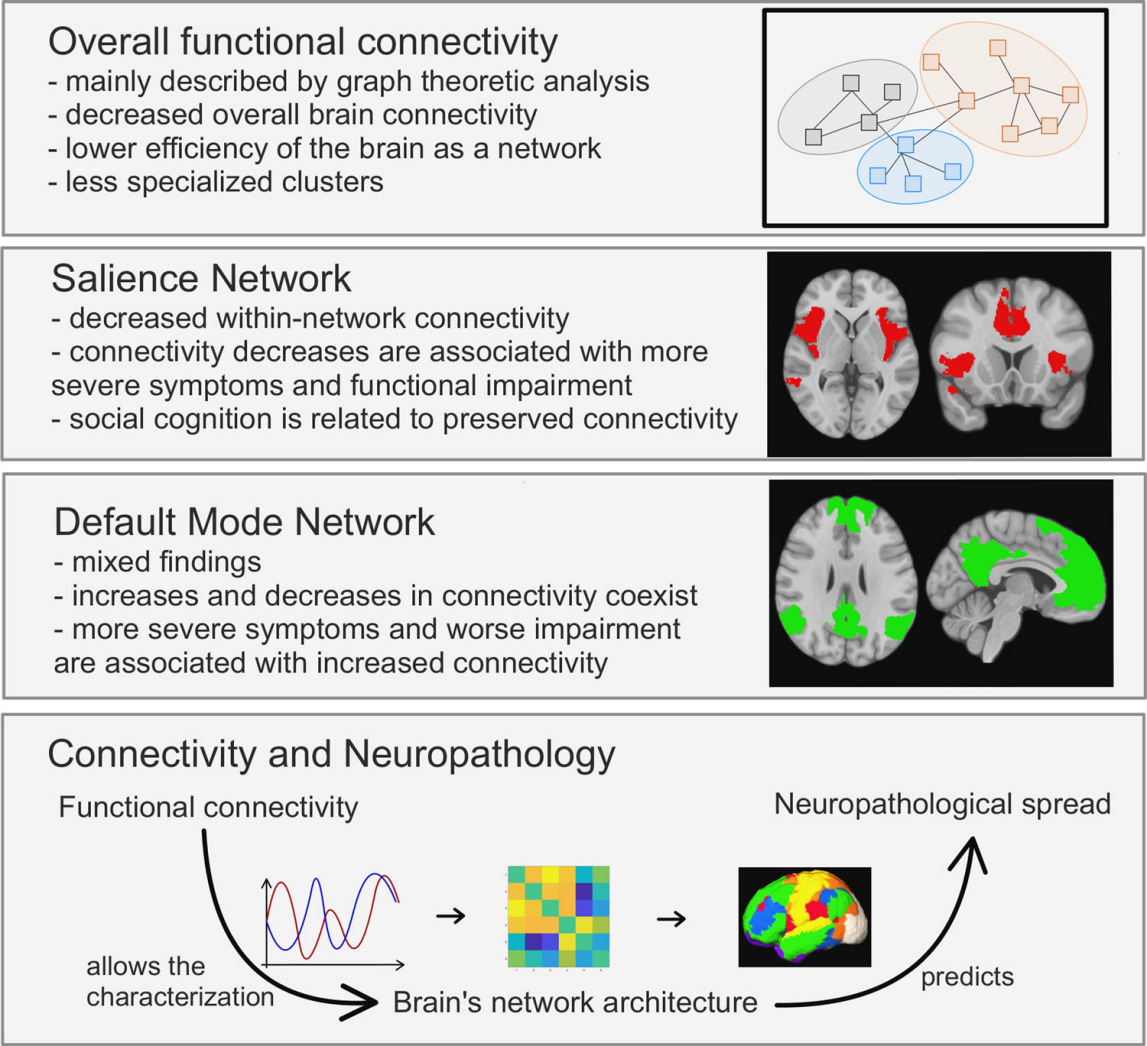
Functional connectivity (FC) in behavioral variant frontotemporal dementia (bvFTD): Graphic summary of the most consistent findings. The illustrations in the last line represent typical steps when analyzing functional connectivity: extraction of time series, calculation of correlations between time series, and identification of sets of functionally connected brain regions. See Section 3.1 for more details. Brain networks were derived from the atlas from Schaefer et al. (2018) using the same tools as in Figure 3

The anterior insula and the SN are the focus of bvFTD‐related changes in FC, further endorsing the view that bvFTD is a disease of the frontoinsula (although a significant proportion of patients may not fall into this category). At the same time, other brain regions and networks also present connectivity changes, such as the DMN. FC is associated with behavioral and cognitive measures in bvFTD. The potential as a biomarker has been endorsed by individual patient classification data. Figure 5 represents a graphic summary of the most consistent findings.

The field is not close to the deployment of FC as a clinical tool, but the next years are expected to bring new, bigger studies aiming at closing this gap. Meanwhile, further methodological advances are expected, and due to the myriad of analytical options one big challenge will be to eventually begin to harmonize procedures. This is true for the broader field of FC (i.e., not specific to bvFTD) which means that progresses may even result from studies of other neuropsychiatric diseases and other branches of neuroscience. Multicentric initiatives with larger samples and incorporation of biomarkers will hopefully lead to more accurate diagnosis and to a framework of dynamic biomarkers that incorporate the clinical, genetic, and neuropathological diversity of bvFTD.

## CONFLICT OF INTEREST

The authors declare no conflict of interest.

### PEER REVIEW

The peer review history for this article is available at: https://publons.com/publon/10.1002/brb3.2790.

## Data Availability

Data sharing not applicable as no new data were generated.
